# Care Deficiencies and Super-Organization of American Nursing Homes in Hospital Referral Region

**DOI:** 10.3389/fpubh.2020.582405

**Published:** 2021-01-20

**Authors:** Tyler Pittman

**Affiliations:** Biostatistics Department, Princess Margaret Cancer Centre, Toronto, ON, Canada

**Keywords:** social network analysis, degree-based centrality, ownership group, total weighted health survey score, registered organization

## Abstract

Super-organization has been associated with worse care quality in nursing homes. Previous research on the chain ownership of American nursing homes excluded government facilities in public-private partnerships, and focused on corporate entities. This longitudinal study proposes a novel method of demarcating the latent ownership networks of for-profit, government and non-profit nursing homes in the United States through use of open data and social network analysis. Facility characteristics and care quality measures were analyzed from an ecological cohort of 9,001 American nursing homes that had a registered organization for owner, and were reimbursed through Medicare or Medicaid. Information was obtained from the Nursing Home Compare open datasets at five semi-annual processing dates from March 2016 to March 2018. Ownership networks of American nursing homes were constructed using the exact legal name of registered organizations. As hospital discharge is a routine admission source of nursing home residents, hospital referral region was actualized to demarcate focal area. Utilizing Bayesian hierarchical models, the association between nursing home super-organization in hospital referral region (inferred by degree-based centrality and Herfindahl-Hirschman Index) to scope of cited care deficiencies (denoted by Total Weighted Health Survey Score) was explored. The percentage of nursing homes having super-organization increased from 56.8 to 56.9% over the 2-year period. During this interval, the mean size of nursing home ownership group in hospital referral region increased from 3.11 to 3.23 facilities. Overall, super-organization in hospital referral region was not associated with care deficiencies in American nursing homes. However, being part of an ownership group with more facilities was beneficial for care quality among nursing homes with super-organization.

## Introduction

Chain affiliation of nursing homes in the United States and the care quality of these facilities has been of concern for the past three decades (1, 2), as well as their super-organization (2). Increased competition among nursing homes for resident admission has been shown to be inversely associated with scope of care deficiency citations (3). However, scholarly work on super-organization has suffered from caveats, such as excluding government nursing homes with shared owners of for-profit nursing homes in public-private partnerships. The traditional method of assessing competition among nursing homes for resident admission makes use of the Herfindahl-Hirschman Index. Use of this metric is common in health sciences literature to denote market competition (4), and is defined as the sum of the squares of the market share ratio by the number of beds for each nursing home in a given locality (2). To account for the chain affiliation of nursing homes in focal areas, a newer derivation of this metric has been proposed that measures the proportion of market concentration due to super-organization, known as the delta Herfindahl-Hirschman Index (4). Prior research has examined the association between nursing home super-organization and care quality in county (2), and American state (5). As hospital discharge is an admission source of nursing home residents (6), hospital referral region (HRR) can be considered as an ecological basis for the focal area.

With the advent of social network analysis, a novel approach is available to determine the super-organization of nursing homes through shared ownership. The current study uncovers the latent ownership networks of nursing homes, through application of a bipartite projection consisting of facilities and owners. Networks utilizing this method summarize the associative ties between two different levels of actors (7). Until recent, the accuracy of nursing home ownership information reported to the Centers for Medicare and Medicaid Services (CMS) has also been questioned, as governments lacked the ability to levy penalties for non-compliance (8). However, progress on name matching has been made through recent revisions enacted in the Uniform Commercial Code (9). Since 2013, registered organizations with property as collateral in the United States have been required to use their exact legal name from the public organic record on statutory documents, or face civil fines (10).

Risk of spurious association is an issue with key matching, as it is conceivable for multiple individuals to have the same name. However, the exact legal names of registered organizations are required to be unique within American states (9), and are trademark enforced within market area for competing business (11). Thus, it is viable to uncover the ownership networks of nursing homes by registered organizations, while excluding nursing homes that are owned solely by individuals. Due to accountability and tax advantages, many owners of nursing homes are organizations incorporated as limited liability companies or real estate trusts (8). Extending a classification posited previously for chain ownership (2), nursing homes that share one or more owners with another nursing home in their hospital referral region can be deemed to have “multiple affiliation.” Those facilities without a shared owner to another nursing home are considered to have “single affiliation.” Multiple affiliation of a nursing home denotes super-organization (2).

Aside from the Herfindahl-Hirschman Index, supplementary measures derived through social network analysis can be used to denote market concentration in a catchment area. An example is degree-based centrality, which quantifies the number of “others” that a given actor has ties with (12). Key players, affiliation patterns and hierarchies can all be discerned from degree-based centrality (13). Regarding corporate acquisitions, research has shown actors to obtain ownership in firms that they wish to influence the practices of, with rival actors in strategic competition imitating this behavior (14). Although an association has been shown between chain affiliation and better care quality of some nursing homes (2), it is unknown how super-organization measures derived from degree-based centrality relate to care quality in American nursing homes.

The current study aimed to explore the association between super-organization of nursing homes and scope of cited care deficiencies. Measures of super-organization that were explored included derivations of the Herfindahl-Hirschman Index, and degree-based centrality from social network analysis. The following research questions were investigated over the study period of March 2016 to March 2018:

- Did nursing homes with super-organization (multiple affiliation) through shared ownership by registered organizations in their HRR have fewer care deficiencies?
- Did the percentage of nursing homes with super-organization increase? Was there a change in the size and number of ownership groups in the United States?
- Was there an association between the mean size of nursing home ownership group per HRR and the scope of care deficiencies?
- Did the increase in market concentration due to super-organization (delta Herfindahl-Hirschman Index) per HRR have an association with scope of care deficiencies?
- Was there considerable variation in the scope of care deficiencies between American states, and HRRs?

## Methods

In this observational study, a two-step analytical procedure was utilized. Social network analysis was performed in the first step to derive variables at the HRR-level, such as the mean size of nursing home ownership group. Measures utilizing the Herfindahl-Hirschman Index were also calculated at the HRR-level. In the second step, statistical analysis was conducted to evaluate the association between variables derived at the HRR-level to scope of cited care deficiencies, while also controlling for facility and resident characteristics of nursing homes. Total Weighted Health Survey Score (TWHSS) was used to denote scope of care deficiencies. This is a metric that is produced by the CMS, and is a weighted count of deficiencies cited from the three most recent cycles of recertification inspection for nursing home, and any complaint inspection in the past year (15).

### Sample and Data Sources

Data at the facility-level of individual nursing homes was linked by “Federal Provider Number” for spreadsheets comprising: Online Survey Certification and Reporting (OSCAR) care deficiencies, Minimum Data Set (MDS) quality measures, penalties, provider characteristics and ownership information from the Nursing Home Compare (NHC) open datasets (16). From the NHC website, datasets containing these measures were obtained at five semi-annual processing dates for the first day of: March 2016, September 2016, March 2017, September 2017, and March 2018. Files were merged by Federal Provider Number for each processing date, and then aggregated by Federal Provider Numbers between processing dates. This sample yielded a cohort of 15,264 licensed nursing homes that could be tracked longitudinally and placed by ZIP code to one of the 306 HRRs bisecting the 50 American states and District of Columbia. Of these 15,264 nursing homes, 9,001 met the study criterion of having ownership by a registered organization, with information for scope of cited care deficiencies and facility characteristics (such as nurse staffing) for each processing date. Prior research has constrained care quality analysis to nursing homes with complete data for these measures (17).

### Social Network Analysis

The igraph package (18) (version 1.2.2) in R (19) (version 3.5.1) was utilized to create networks having two levels of actors within focal areas corresponding to the 306 HRRs in the United States. These levels comprised nursing home facilities and nursing home owners in a bipartite projection, denoted by the “Owner Name” and “Federal Provider Number” fields from the NHC datasets. To facilitate key matching in the creation of these networks, all commas, periods and multiple spacing between characters for records obtained from the Owner Name field were removed (20). From the bipartite projection, explanatory variables at the HRR-level were derived and are discussed in a following section. To determine the ownership groups of nursing homes by registered organizations, the Louvain modularity algorithm was applied for community detection (21).

### Statistical Analyses

Hierarchical modeling was employed using the MCMCglmm package (22) (version 2.26) in R (version 3.5.1). A Bayesian analysis of the Poisson random effects model was utilized with repeated measures. This framework was chosen, as it is flexible for over-dispersed case counts and making numerical inference for data not obtained through a random sample (22). To adhere to a Poisson distribution, values of cited care deficiencies were discretized to the nearest integer. Two Bayesian hierarchical models (Models 1 and 2) were formulated. This was required to prevent multicollinearity between distinct resident quality measures in the MDS (Model 2), and the Five-Star Quality Measure derived from them (Model 1) (15).

Prevalence ratios (PRs) were calculated from the exponentiated coefficients in the Bayesian hierarchical models (23), and were the measure of inference. This quantity is interpreted as the percentage change in care deficiencies resulting from a unit change in a continuous explanatory variable, or of disparate classification levels to the reference level for a categorical variable. Mean-centering of continuous explanatory variables was performed. To account for cross-classification (24), the random effects of HRR and American state were fitted additively in the models. Non-informative priors were specified to generate robust estimates of model parameters in the posterior distribution (24). A sampling phase of 115,000 iterations with a burn-in of 15,000 iterations, and a thinning interval of 10 was specified to obtain 10,000 samples in the posterior distribution. To infer statistical differences in hypothesis testing, the highest posterior density interval (HPDI) was utilized, and is similar to a confidence interval (25). The proportion of variation explained in outcome that is attributable to random effect was deduced from the intraclass correlation coefficient (ICC).

### Measures Denoting Super-Organization in Focal Areas

Five explanatory variables denoting super-organization were derived through social network analysis at the focal area level, corresponding to HRR. Respectively, these constitute: prevalence of nursing homes in super-organization (multiple affiliation) per HRR, mean size of nursing home ownership group per HRR, Herfindahl-Hirschman Index, affiliation-accounted Herfindahl-Hirschman Index and the delta Herfindahl-Hirschman Index.

The Herfindahl-Hirschman Index (HHI) is a measure of nursing home competition within a focal area (2). The derivation of the HHI among n, nursing homes located in a HRR at a specified time point is as follows:


(1) $$\begin{array}{r} HHI=\\ \overset{\text{n}}{\underset{\text{i}=1}{\sum }}{\left[\frac{\text{total number of certified beds in nursing home},\text{ i}}{\text{total number of certified beds in HRR}}\right]}^{2} \end{array}$$

Similar to the chain-accounted Herfindahl-Hirschman Index (4), the affiliation-accounted Herfindahl-Hirschman Index (AHHI) accounts for the ownership networks of nursing homes with shared ownership by a registered organization. This metric is always equivalent to or greater than the HHI. The computation of the AHHI among n, nursing homes located in a HRR is:


(2) $$\begin{array}{r} AHHI=\overset{\text{n}}{\underset{\text{i}=1}{\sum }}{\left[\frac{\text{total number of certified beds in affiliated nursing home group},\text{ i}}{\text{total number of certified beds in HRR}}\right]}^{2} \end{array}$$

The delta Herfindahl-Hirschman Index (HHI) is the difference between the AHHI and the HHI (4). This denotes the increase in the proportion of market concentration per HRR that arises purely from the super-organization of nursing homes.

### Facility and Resident Characteristic Measures in Nursing Homes

Explanatory variables at the nursing home-level were examined for their association to care deficiencies. Adjusted nurse staffing hours per resident day (HRD) is a metric produced by the CMS that adjusts for case-mix (8, 15), and was analyzed for certified nursing assistant, licensed practical nurse and registered nurse. Facility characteristics of nursing homes were also fitted in the models. These included: number of certified beds, occupancy ratio, years in business, ownership type (for-profit, government, non-profit), hospital location (no, yes), special focus facility (no, yes), continuing care retirement community (no, yes), resident or family council (no, yes), and if ownership changed in the past year (no, yes). The Five-Sar Quality Measure (Model 1) and distinct resident characteristics by measure code from the MDS (Model 2) were also fitted, although in separate models to reduce multicollinearity. Effect modification between ownership type and affiliation class of nursing home was also explored.

## Results

### Overview

**Figure 1:**
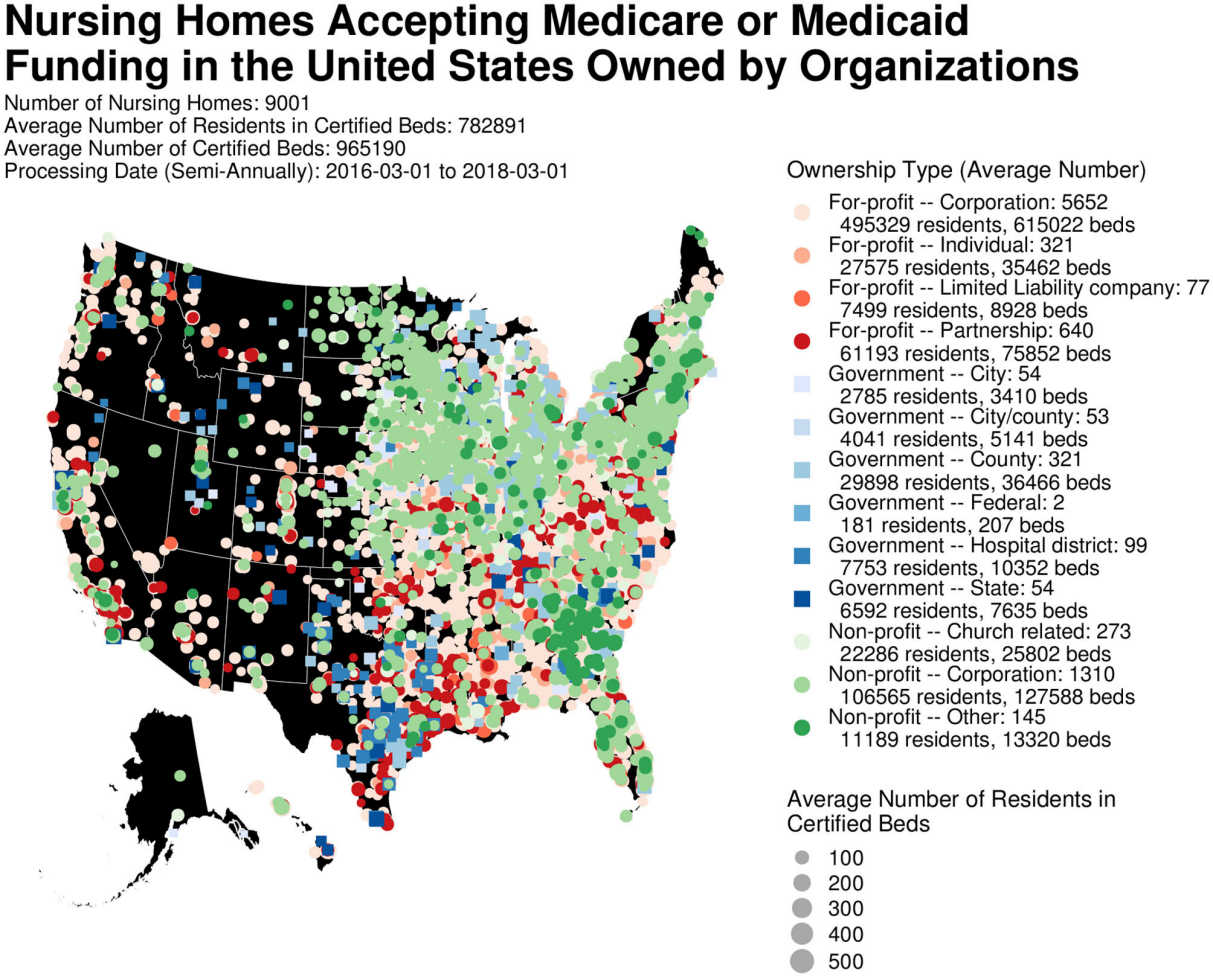
Nursing homes accepting Medicare or Medicaid reimbursement with a registered organization for owner. Figure was created using R version 3.5.1.

Complete longitudinal study information for facility characteristics and scope of care deficiencies was available for 9,001 nursing homes having a registered organization as owner (Figure 1; Table 1). Since many nursing homes had missing information for resident characteristics from the MDS for one or more processing date, the sample size of nursing homes for Model 2 is smaller than that for Model 1 (6,693 vs. 9,001). Of the 306 HRRs in the United States (26), each contained at least one nursing home that had a registered organization for owner. However, the number of HRRs represented decreased to 294 for the sample of 9,001 nursing homes with complete information for each of the five semi-annual processing dates in Model 1, and to 293 HRRs for the sample of 6,693 nursing homes in Model 2.

**Table 1:** Prevalence ratios of total weighted health survey score for Models 1 and 2.

	**Model 1 (NHs** **=** **9,001; HRRs** **=** **294)**				**Model 2 (NHs** **=** **6,693; HRRs** **=** **293)**			
**Variable**	**PR**	**l-95 HPDI**	**u-95 HPDI**	**MCMC p**	**PR**	**l-95 HPDI**	**u-95 HPDI**	**MCMC p**
Nurse staffing								
Adjusted CNA staffing (HRD)	0.967	0.956	0.980	<0.001	0.963	0.950	0.977	<0.001
Adjusted LPN staffing (HRD)	1.013	0.998	1.030	0.102	1.010	0.991	1.028	0.290
Adjusted RN staffing (HRD)	0.795	0.770	0.820	<0.001	0.822	0.789	0.854	<0.001
Facility characteristics								
Number of residents in certified beds (n)	1.003	1.002	1.003	<0.001	1.003	1.002	1.003	<0.001
Occupancy ratio (%)	0.995	0.994	0.995	<0.001	0.995	0.994	0.996	<0.001
Years in business (n)	1.006	1.004	1.007	<0.001	1.004	1.003	1.006	<0.001
Ownership type								
For-profit	Ref				Ref			
Government	0.954	0.909	0.999	0.047	0.950	0.901	0.997	0.045
Non-profit	0.878	0.845	0.911	<0.001	0.893	0.859	0.930	<0.001
Provider resides in hospital								
No	Ref				Ref			
Yes	0.914	0.861	0.972	0.004	1.116	1.021	1.213	0.010
Special focus facility								
No	Ref				Ref			
Yes	1.855	1.740	1.993	<0.001	1.836	1.711	1.969	<0.001
Continuing care retirement community								
No	Ref				Ref			
Yes	0.914	0.884	0.948	<0.001	0.941	0.906	0.977	0.002
Has a resident or family council								
No	Ref				Ref			
Yes	1.073	1.030	1.120	0.001	1.010	0.958	1.064	0.712
Provider changed ownership in past year								
No	Ref				Ref			
Yes	0.992	0.967	1.016	0.549	0.990	0.963	1.016	0.457
Five-Star Quality Measure from MDS								
Category 1	Ref							
Category 2	0.959	0.940	0.977	<0.001				
Category 3	0.936	0.917	0.955	<0.001				
Category 4	0.899	0.881	0.919	<0.001				
Category 5	0.839	0.821	0.859	<0.001				
Derived from social network analysis at HRR-Level								
Prevalence of NHs in multiple affiliation	0.999	0.997	1.000	0.026	0.999	0.997	1.000	0.084
Mean size of nursing home ownership group	0.983	0.975	0.992	<0.001	0.978	0.969	0.987	<0.001
Delta Herfindahl-Hirschman Index	1.032	0.655	1.635	0.898	1.082	0.668	1.780	0.759
Overall ownership network class by HRR								
Multiple affiliation	Ref				Ref			
Single affiliation	1.022	0.995	1.049	0.103	1.030	1.002	1.058	0.037
Interaction of ownership type by affiliation class								
For-profit and multiple affiliation	Ref				Ref			
Government and single affiliation	0.885	0.822	0.951	<0.001	0.890	0.818	0.969	0.008
Non-profit and single affiliation	0.965	0.919	1.014	0.159	0.986	0.933	1.041	0.612
Resident characteristics by measure code								
Long-stay prevalence, four quarter averages (%)								
401 – Need for help with ADLs has increased					1.006	1.005	1.008	<0.001
402 – Self-report moderate to severe pain					1.001	1.000	1.003	0.135
403 – Have pressure ulcers					1.013	1.011	1.016	<0.001
404 – Lose too much weight					1.002	1.000	1.004	0.019
405 – Lose control of bowels or bladder					0.999	0.998	1.000	0.003
406 – Catheter inserted and left in bladder					1.007	1.003	1.011	<0.001
407 – Have urinary tract infection					0.997	0.995	1.000	0.034
408 – Have depressive symptoms					1.000	0.999	1.001	0.914
409 – Were physically restrained					1.006	1.000	1.012	0.072
410 – Experienced fall with major injury					1.004	1.001	1.007	0.007
411 – Given seasonal influenza vaccine					0.997	0.996	0.998	<0.001
415 – Given pneumococcal vaccine					0.998	0.997	0.999	0.001
419 – Received an anti-psychotic medication					1.005	1.003	1.006	<0.001
451 – Ability to move independently worsened					1.001	0.999	1.002	0.431
452 – Received an anti-anxiety medication					1.000	0.999	1.002	0.465
Short-stay prevalence, four quarter averages (%)								
424 – Self-report moderate to severe pain					1.002	1.001	1.003	0.002
425 – Have pressure ulcers that are new					1.008	1.002	1.013	0.009
426 – Given seasonal influenza vaccine					0.998	0.998	0.999	<0.001
430 – Given pneumococcal vaccine					0.998	0.997	0.998	<0.001
434 – Received an anti-psychotic medication					1.009	1.006	1.013	<0.001
471 – Made improvements in function					0.998	0.997	0.999	<0.001

*PR, prevalence ratio; HPDI, highest posterior density interval; MCMC, Markov chain Monte Carlo; HRR, hospital referral region; HRD, hours per resident day; ADLs, activities of daily living; NH, nursing home*.

Of the 9,001 nursing homes, 6,690 (74.3%) were for-profit, 583 (6.5%) were government-owned and 1,728 (19.2%) were non-profit (Figure 1). From the mean population of 782,891 residents in certified beds over the 2-year study period, 591,596 (75.6%) were housed in for-profit nursing homes, 140,040 (17.9%) in non-profit and 51,253 (6.6%) in government facilities (Figure 1).

### Ownership Network

**Figure 2:**
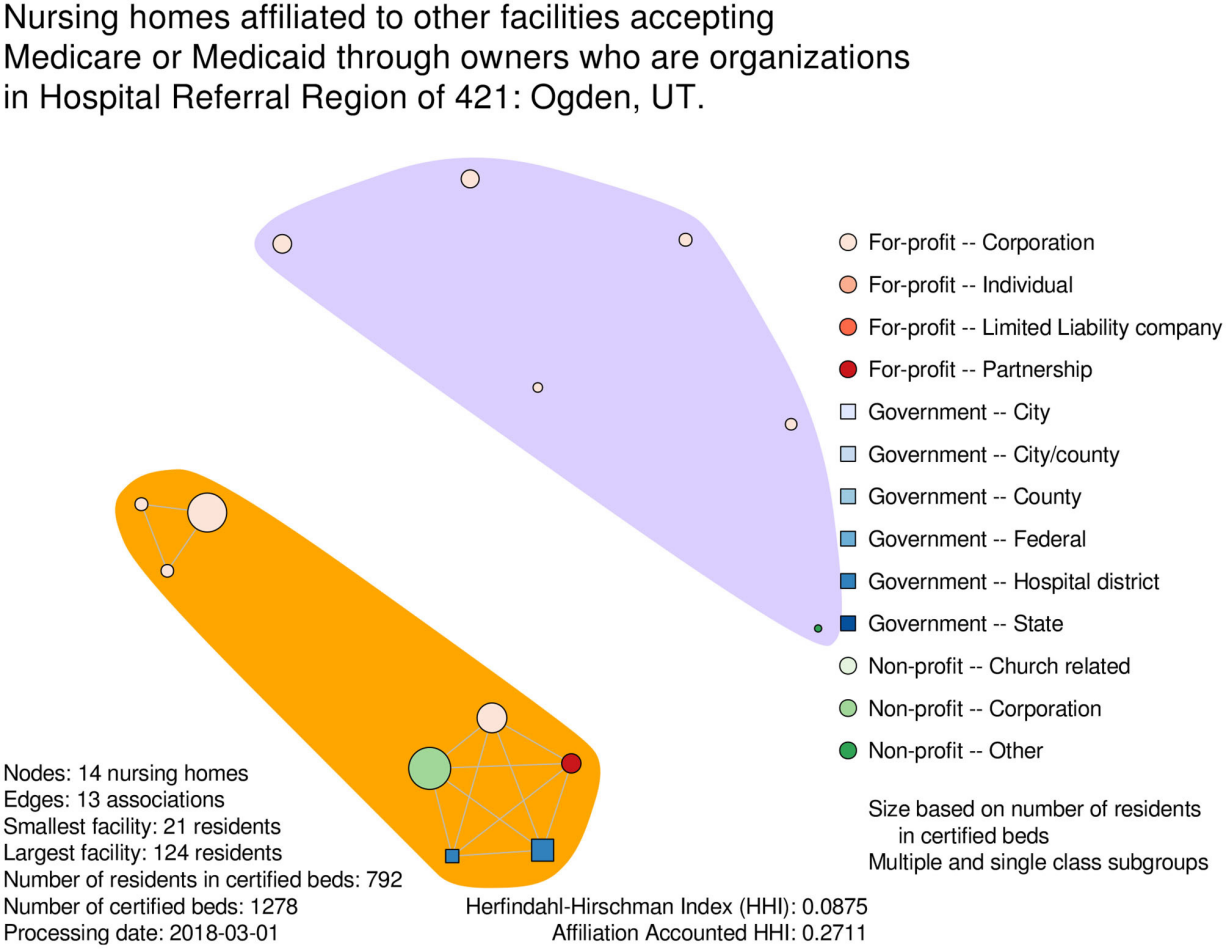
Exemplar graph diagram of nursing home super-organization in a hospital referral region. Figure was created using R version 3.5.1.

Graph diagrams were formulated for each of the 294 HRRs that contained a nursing home with a registered organization for owner, at each of the five semi-annual processing dates. A visual representation of super-organization with regard to the processing date of March 1, 2018 is presented in Figure 2 for nursing homes in the HRR of Ogden, Utah. In this graph diagram, six facilities had no shared ownership by a registered organization to another nursing home in this focal area, while eight did. Those nursing homes with shared ownership by a registered organization to another facility were deemed to have “multiple affiliation” or super-organization. Two ownership groups among nursing homes with super-organization were evident. The larger of the ownership groups was comprised of for-profit, government and non-profit facilities, with each nursing home in this group having ownership by a shared organization. Although the HHI of 0.088 implies a market with healthy competition, the AHHI of 0.271 suggests higher concentration when adjusting for super-organization.

### Model 1

Prevalence ratios of care deficiencies with regard to explanatory variables (including the Five-Star Quality Measure) are presented in Table 1. With regard to nurse staffing, each additional hour per resident day (HRD) above the overall average for certified nursing assistants was associated with 3.3% fewer (adjusted PR: 0.967; 95% HPDI: 0.956–0.980) care deficiencies. A larger 20.5% decrease (adjusted PR: 0.795; 95% HPDI: 0.770–0.820) in care deficiencies was observed for each hour increase in registered nurse staffing per resident day. Care deficiencies increased by 0.3% (adjusted PR: 1.003; 95% HPDI: 1.002–1.003) for each additional resident in nursing home size. However, each percentage increase in occupancy ratio was associated with 0.5% fewer (adjusted PR: 0.995; 95% HPDI: 0.994–0.995) care deficiencies. Nursing homes in business for longer had a higher scope of care deficiencies on average, with a 0.6% increase (adjusted PR: 1.006; 95% HPDI: 1.004–1.007) for each additional year.

In comparison to for-profit facilities, care deficiencies among government nursing homes were 4.6% lower (adjusted PR: 0.954; 95% HPDI: 0.909–0.999); while care deficiencies among non-profit nursing homes were 12.2% lower (adjusted PR: 0.878; 95% HPDI: 0.845–0.911), respectively. Hospital-based nursing homes had 8.6% fewer (adjusted PR: 0.914; 95% HPDI: 0.861–0.972) care deficiencies than non-hospital-based facilities, on average. Special focus facilities had almost 1.9 times as many (adjusted PR: 1.855; 95% HPDI: 1.740–1.993) care deficiencies than non-special focus facilities. Regarding the Five-Star Quality Measure derived from the MDS, consecutive increases in ordinal category were associated with fewer care deficiencies in nursing homes.

Measures denoting super-organization and their association to scope of care deficiencies are also presented in Table 1. A 0.1% decrease (adjusted PR: 0.999; 95% HPDI: 0.997–1.000) in care deficiencies was observed for every percentage increase in the prevalence of nursing homes with multiple affiliation (super-organization) in their HRR. For each facility increase in the size of nursing home ownership group per HRR, a 1.7% decrease (adjusted PR: 0.983; 95% HPDI: 0.975–0.992) in care deficiencies was observed. Examining the effect modification between ownership type and super-organization, government nursing homes without a shared owner to another facility in their HRR had 11.5% fewer (adjusted PR: 0.885; 95% HPDI: 0.822–0.951) care deficiencies than for-profit facilities with a common owner to another nursing home in their HRR.

### Model 2

Results obtained from Model 2 are similar to Model 1, with the addition of aggregated long-stay and short-stay resident characteristics by quality measure code from the MDS and the exclusion of the Five-Star Quality Measure (Table 1). For every percentage increase in the prevalence of residents whose help with activities of daily living had increased, care deficiencies increased by 0.6% (adjusted PR: 1.006; 95% HPDI: 1.005–1.008) on average in nursing home. Indicative of an adverse event, each percentage increase in the prevalence of residents with pressure ulcers was associated with a 1.3% increase (adjusted PR: 1.013; 95% HPDI: 1.011–1.016) in care deficiencies. A higher than average prevalence of residents who experienced one or more falls with major injury was associated with more care deficiencies (adjusted PR: 1.004; 95% HPDI: 1.001–1.007) in nursing home. A protective effect was shown in the prevalence of residents given the seasonal influenza vaccine, with every percentage increase above the mean being associated with 0.3% fewer (adjusted PR: 0.997; 95% HPDI: 0.996–0.998) care deficiencies in nursing home.

### Summary of Random Effects at the HRR-Level

Descriptive statistics of explanatory variables derived at the HRR-level through social network analysis are presented by processing date in Table 2. Consecutive increases in Total Weighted Health Survey Score was observed over the study period, ranging from 54.5 in March 2016 to 63.6 by March 2018. The prevalence of nursing homes with super-organization (multiple affiliation) in their HRR was over 56% for each processing date. As scope of cited care deficiencies followed a Poisson distribution, values for the standard deviation can (and did) exceed the mean. From March 2016 to March 2018, the mean size of nursing home ownership group per HRR increased from 3.11 to 3.23 facilities. The overall HHI was 0.07 for each processing date. This denotes the increase in the proportion of market concentration among American nursing homes in HRR hat was attributable to super-organization.

**Table 2:** HRR-level fixed effect characteristics by processing date (Model 1); intraclass correlation coefficients for the random effects (Models 1 and 2).

	**Processing Date** **(*****n*** **=** **5)**									
	**March 2016**		**September 2016**		**March 2017**		**September 2017**		**March 2018**	
**Variable**	***M*/%/*n***	**SD**	***M*/%/*n***	**SD**	***M*/%/*n***	**SD**	***M*/%/*n***	**SD**	***M*/%/*n***	**SD**
Total Weighted Health Survey Score of 9,001 NHs in **Model 1** (*n*)	54.48	60.41	56.82	65.36	58.99	70.61	60.55	71.66	63.55	80.61
Derived by social network analysis at HRR-level (*n* = 294) of 9,001 NHs in **Model 1**										
Prevalence of NHs in multiple affiliation (%)	56.76	16.66	56.99	16.67	56.86	16.98	56.73	16.54	56.91	16.36
Mean size of NH ownership group (n)	3.11	2.20	3.14	2.20	3.18	2.26	3.22	2.30	3.23	2.36
Herfindahl-Hirschman Index	0.06	0.05	0.06	0.05	0.06	0.04	0.06	0.04	0.06	0.04
Affiliation Herfindahl-Hirschman Index	0.13	0.08	0.13	0.08	0.13	0.08	0.13	0.08	0.13	0.08
Delta Herfindahl-Hirschman Index	0.07	0.05	0.07	0.05	0.07	0.06	0.07	0.06	0.07	0.06
Total number of NHs owned by organizations	10728		10875		11009		10736		10943	
From NHs in multiple affiliation per HRR										
Total number of NHs with multiple affiliation	6940		7083		7191		7058		7146	
Total number of organization owners	17202		17289		18847		18271		18839	
Total number of ownership groups	1870		1896		1905		1865		1878	
	**Model 1** **(*****n*** **=** **9,001)**			**Model 2** **(*****n*** **=** **6,693)**						
	**ICC**	**l-95**	**u-95**	**ICC**	**l-95**	**u-95**				
**Variable**		**HPDI**	**HPDI**		**HPDI**	**HPDI**				
HRR-level										
Random intercept	0.073	0.042	0.103	0.081	0.044	0.108				
American State-level										
Random intercept	0.176	0.097	0.282	0.193	0.098	0.293				

*M, mean; SD, standard deviation; HRR, hospital referral region; NH, nursing home*.

*ICC, intraclass correlation coefficient; HPDI, highest posterior density interval; HRR, hospital referral region*.

Summary information regarding the ownership groups from Model 1 is shown (Table 2). When aggregated by HRR, the number of registered organizations in the United States that had shared ownership of a nursing home among one or more “other” registered organizations increased from 17,202 at the start of study, to 18,839 by the end. These registered organizations comprised 1,870 ownership groups in March 2016 and 1,878 ownership groups in March 2018.

Table 2 also presents the ICCs for the additive random effects of American state and HRR that were obtained from Models 1 and 2. The proportion of the total variation in scope of cited care deficiencies attributable to American state after controlling for HRR was approximately 17.6% in Model 1, and 19.3% in Model 2. Conversely, the proportion of total variation in care deficiencies that was explained by HRR after controlling for American state was 7.3% in Model 1, and 8.1% in Model 2.

### Geographical Presentation of Random Effects

**Figure 3:**
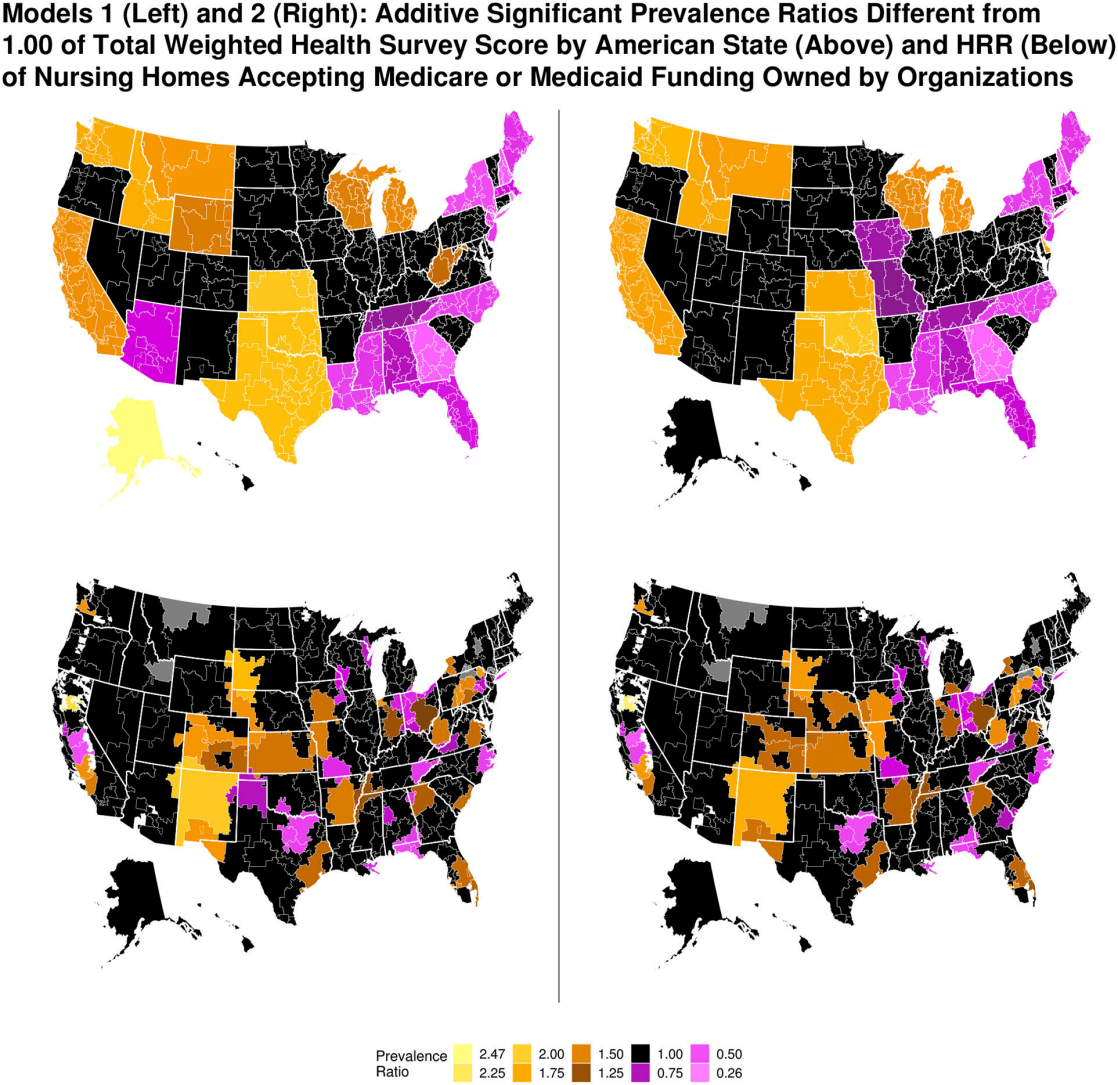
Additive prevalence ratios of total weighted health survey score by American state and hospital referral region for Models 1 and 2. Maps were generated by author. Figure was created using R version 3.5.1.

Prevalence ratios of the random effects are displayed geographically (Figure 3). Those of American state are discussed first. Examining Model 1, the American states of Alaska, Washington, California, Idaho, Montana, Kansas, Oklahoma, Texas, Wisconsin, Michigan and West Virginia each had a prevalence ratio that was respectively higher than the national average for scope of cited care deficiencies. Nursing homes with the highest care deficiencies were found in Alaska, having a prevalence ratio almost two and a half times higher than the national average. Nursing homes in Rhode Island had the fewest care deficiencies, at almost three-quarters less than the national average.

Prevalence ratios by HRR are also presented in Figure 3. Localities in the United States outside the boundaries of any HRR are shown with an absence of color; gray denotes HRRs that were not included in the present study. With regard to Model 1, some HRRs with a higher prevalence ratio than the national average for scope of care deficiencies were located in American states that also had a higher prevalence ratio of care deficiencies than the national average. An example is Chico, California. Other HRRs had a lower prevalence ratio for care deficiencies than the national average, but were located in American states with a higher than average prevalence ratio, such as Modesto, California. The converse was also possible, such as the HRR comprising Atlanta, Georgia. Geographical findings in Model 2 were similar to Model 1.

## Discussion

Super-organization of licensed nursing homes through common ownership by registered organizations was found to be an intensifying process, as corroborated by the current study that accounted for latent ownership networks. The prevalence of American nursing homes having super-organization in their HRR increased by three twentieths from 2016 to 2018. As hypothesized, nursing homes with shared ownership by a registered organization to another facility in their HRR had a lower scope of care deficiencies than nursing homes without a common owner. An explanation for this could be due to the uptake of improved practices through knowledge transfer among owners (27). Interestingly, the number of ownership groups increased by roughly 1% from 2016 to 2017, and then decreased by 1% to 2018. Nursing homes that were part of a larger ownership group with more facilities had fewer care deficiencies. This relationship was observed in another study with regard to nursing home chains (2).

No association was established between the increase in market concentration that was due to super-organization (HHI) and scope of cited care deficiencies. However, at 0.07, the magnitude of the overall HHI was relatively small. Although this difference was greater than the 0.02 observed between chain-accounted HHIs and unadjusted HHIs of American nursing homes in another study, that used the county-level as a focal area (4). The overall affiliation-accounted Herfindahl-Hirschman Index (AHHI) of 0.13 among American nursing homes in HRR from the current study was much less than the overall chain-accounted HHI of 0.55 observed in the prior study (4). An explanation for this is that market competition of nursing homes is much less concentrated per HRR in comparison to county, which makes sense as HRRs are generally larger by both population and geographical area. A limitation of the current study is that ownership networks are based on the integrity of registered names in the NHC datasets, with market concentration likely being underestimated. Additionally, direct comparisons cannot be made on the nature of market concentration within HRR to county, as the current study used a more encompassing definition of super-organization than the characterization of chain affiliation utilized by previous studies.

Effect modification between ownership type and super-organization of nursing home was shown in the association to care deficiencies. As expected, government facilities without a shared owner to another nursing home in their HRR had a lower scope of care deficiencies than for-profit nursing homes with super-organization. However, no difference in scope of care deficiencies was observed between non-profit nursing homes without super-organization and for-profit nursing homes with super-organization in their HRR. This suggests that super-organization is more beneficial for non-profit than government nursing homes. Regardless of super-organization, government and non-profit nursing homes in general had a lower scope of cited care deficiencies than for-profit nursing homes over the study period. This could be due to a variety of factors, such as profit-seeking behaviors that diminished clinical care, and better adherence to protocol (and enforcement of penalties arising from inspection) among government and non-profit facilities (28).

Many of the findings presented in this study that associate facility and resident characteristics to care deficiencies concur with results in the literature. High levels of registered nurse and certified nursing assistant staffing were negative predictors for care deficiency count (17). With regard to facility characteristics, special focus facilities had more care deficiencies. This is not unexpected, as that designation is for providers with among the worst care quality as reported from previous inspection (15). On the converse, continuing care retirement community were associated with fewer care deficiencies. Typically, these facilities are populated by residents with greater incomes (29). With regard to resident characteristics, nursing homes containing residents with a higher prevalence of pressure ulcers had more care deficiencies (17). Seasonal influenza and pneumococcal vaccinations of residents offered a preventative effect to care deficiencies in nursing home, although vaccine implementation as a standard operating procedure may be more prominent in non-profit and government facilities (30).

As a limitation, the current study did not consider the lagged effect of organization changes in nursing home ownership to care quality. However, an indicator which denoted if provider changed ownership in the previous year from the NHC datasets was included for analysis. Lagged effects should be considered in studies with longer follow-up periods. A previous study ascertained that nursing homes acquired by the largest for-profit chains in the United States had more care deficiency citations in the subsequent 2 years after acquisition (1). Another study of panel data over a 6-year period found care quality to increase in later years for some independent nursing homes, following acquisition by a for-profit chain (2). In interpreting associations, one should be cautious of the ecological fallacy. In particular, the effect of super-organization to care quality for individual nursing homes may be different than the overall group effect. It is conceivable that there may be for-profit nursing homes in small ownership networks that provide superior care to non-profit or government nursing homes, and are still partially reimbursed by Medicare or Medicaid. An example being luxury care homes with higher staffing levels (29).

Unexpectedly, the mean scope of cited care deficiencies in nursing homes increased considerably over the study period. The reasoning for this is difficult to explain, but could be due to changes in the recertification inspection process enacted by states (15). The importance of locality must be stated, as the ICCs of American state and HRR suggest that large amounts of variation in care deficiencies were explained by these random effects. Considerable variation was also exhibited in the magnitude of care deficiencies across the United States, with states in the South and New England regions, in particular, having a lower prevalence ratio in comparison to the American average. Contextual effects within these administrative units have a large impact on care quality. Examples of such aspects include variation in the minimum threshold of registered nurse HRD staffing levels (31), Medicaid reimbursement rate (32), and discrimination due to race (3). As these factors can be addressed through regulation and enforcement, legislators have a responsibility to actualize care equity in American nursing homes.

## Conclusions

The present study on the effect of nursing home super-organization to care deficiencies, as demarcated by ownership networks of registered organizations, yielded associations in agreement to those from previous studies. Degree-based centrality measures that were derived from social network analysis, such as the mean size of nursing home ownership group, were better associated with care quality than the Herfindahl-Hirschman Index in hospital referral region. In summary, nursing homes with super-organization in hospital referral region that were part of a larger ownership group with more facilities had fewer care deficiencies than nursing homes with super-organization in small ownership groups. The prevalence of American nursing homes with super-organization is increasing gradually.

## Data Availability Statement

Publicly available datasets were analyzed in this study. This data can be found here: https://data.medicare.gov/data/nursing-home-compare.

## Author Contributions

TP conceptualized the manuscript, reviewed appropriate literature, created the figures, performed the analysis, contributed to the article and approved the submitted version.

## Conflict of Interest

The author declares that the research was conducted in the absence of any commercial or financial relationships that could be construed as a potential conflict of interest.

## References

[1] Harrington C Olney B Carrillo H Kang T. Nurse staffing and deficiencies in the largest for-profit nursing home chains and chains owned by private equity companies. Heal Serv Res. (2012) 47:106–28. 10.1111/j.1475-6773.2011.01311.x 22091627 PMC3447240

[2] Banaszak-Holl J Berta WB Bowman M Baum JAC Mitchell W. The rise of human service chains: antecedents to acquisitions and. Manag Decis Econ. (2002) 23:261–82. 10.1002/mde.1065

[3] Grabowski DC. The admission of blacks to high-deficiency nursing homes. Med Care. (2004) 42:456–64. 10.1097/01.mlr.0000124307.17380.df 15083106

[4] Hirth RA Zheng Q Grabowski DC Stevenson DG Intrator O Banaszak-Holl J. The effects of chains on the measurement of competition in the nursing home industry. Med Care Res Rev. (2019) 76:315–36. 10.1177/1077558717701771 29148340 PMC13159488

[5] Swan J Newcomer R. Residential care supply, nursing home licensing, and case mix in four States. Heal Care Financ Rev. (2000) 21:203–29. Available online at: http://search.proquest.com/docview/196950286/11481756 PMC4194680

[6] Goodwin J Li S Zhou J Graham J Karmarkar A Ottenbacher K. Comparison of methods to identify long term care nursing home residence with administrative data. BMC Health Serv Res. (2017) 17:1–8. 10.1186/s12913-017-2318-9 28558756 PMC5450097

[7] Cranmer SJ Desmarais BA. Inferential network analysis with exponential random graph models. Polit Anal. (2011) 19:66–86. 10.1093/pan/mpq037

[8] Harrington C Ross L Kang T. Hidden owners, hidden profits, and poor nursing home care: a case study. Int J Heal Serv. (2015) 45:779–800. 10.1177/0020731415594772 26159173

[9] Hodnefield P. Compliance is key with new UCC debtor-name requirements. RMA J. (2013) 96:76–80.

[10] Weissman M. The Name game: proposed amendments to article 9 of the UCC. RMA J. (2011) 94:40–4.

[11] Mosier G Jackman J. Personal Jurisdiction: Is Internet Presence Enough? J Acad Mark Sci. (1998) 26:164.

[12] Marsden PV. Egocentric and sociocentric measures of network centrality. Soc Networks. (2002) 24:407–22. 10.1016/S0378-8733(02)00016-3

[13] Ortiz-Pelaez A Pfeiffer DU Soares-Magalhaes RJ Guitian FJ. Use of social network analysis to characterize the pattern of animal movements in the initial phases of the 2001 foot and mouth disease (FMD) epidemic in the UK. Prev Vet Med. (2006) 76:40–55. 10.1016/j.prevetmed.2006.04.007 16769142

[14] Haunschild PR. Interorganizational Imitation: the impact of interlocks on corporate acquisition activity. Adm Sci Q. (1993) 38:564–92. 10.2307/2393337

[15] Centers for Medicare and Medicaid Services. Design for Nursing Home Compare Five-Star Quality Rating System: Technical Users' Guide January 2017. (2017). Available online at: https://www.cms.gov/Medicare/Provider-Enrollment-and-Certification/CertificationandComplianc/Downloads/usersguide.pdf (accessed June 1, 2020).

[16] Centers for Medicare and Medicaid Services. Nursing Home Compare Datasets. (2018). Available online at: https://data.medicare.gov/data/nursing-home-compare (accessed June 1, 2020).

[17] Harrington C Zimmerman D Karon SL Robinson J Beutel P. Nursing home staffing and its relationship to deficiencies. J Gerontol Ser B. (2000) 55:S278–87. 10.1093/geronb/55.5.S278 10985299

[18] Csardi G Nepusz T. The igraph software package for complex network research. Int J Comp Sys. (2006)1695:1–9.

[19] R Core Team. R: A Language and Environment for Statistical Computing. (2018). Available online at: http://www.r-project.org/ (accessed June 1, 2020).

[20] US Government Accountability Office. GAO-10-710 Nursing Homes: Complexity of Private Investment Purchases Demonstrates Need for CMS to Improve the Usability and Completeness of Ownership Data. (2010). p. 1–69. Available online at: https://www.gao.gov/products/GAO-10-710 (accessed June 1, 2020).

[21] Blondel VD Guillaume J-L Lambiotte R Lefebvre E. Fast unfolding of communities in large networks. J Stat Mech Theory Exp. (2008) 2008:1–12. 10.1088/1742-5468/2008/10/P10008

[22] Hadfield JD. MCMC methods for multi-response generalized linear mixed models: the MCMCglmm R package. J Stat Softw. (2010) 33:1–22. 10.18637/jss.v033.i0220808728

[23] Zocchetti C Consonni D Bertazzi PA. Relationship between prevalence rate ratios and odds ratios in cross-sectional studies. Int J Epidemiol. (1997) 26:220–3. 10.1093/ije/26.1.220 9126523

[24] Li B Lingsma HF Steyerberg EW Lesaffre E. Logistic random effects regression models: a comparison of statistical packages for binary and ordinal outcomes. BMC Med Res Methodol. (2011) 11:1–11. 10.1186/1471-2288-11-77 21605357 PMC3112198

[25] Hadfield J. MCMCglmm Course Notes. (2018). Available online at: https://cran.r-project.org/web/packages/MCMCglmm/vignettes/CourseNotes.pdf (accessed June 1, 2020).

[26] Dartmouth Medical School. The Dartmouth atlas of health care 1998/the Center for the Evaluative Clinical Sciences. Chicago: American Hospital Publishing, Inc. (1998). 36758135

[27] Ter Wal ALJ Boschma RA. Applying social network analysis in economic geography: framing some key analytic issues. Ann Reg Sci. (2009) 43:739–56. 10.1007/s00168-008-0258-3

[28] Harrington C Woolhandler S Mullan J Carrillo H Himmelstein DU. Does investor ownership of nursing homes compromise the quality of care? Am J Public Health. (2001) 91:1452–5. 10.2105/AJPH.91.9.1452 11527781 PMC1446804

[29] Aaronson WE Zinn JS Rosko MD. Do For-Profit and Not-for-Profit Nursing Homes Behave Differently? Gerontologist. (1994) 34:775–86. 10.1093/geront/34.6.775 7843607

[30] Shefer A McKibben L Bardenheier B Bratzler D Roberts H. Characteristics of long-term care facilities associated with standing order programs to deliver influenza and pneumococcal vaccinations to residents in 13 states. J Am Med Dir Assoc. (2005) 6:97–104. 10.1016/j.jamda.2004.12.020 15871883

[31] Kim H Kovner C Harrington C Greene W Mezey M. A panel data analysis of the relationships of nursing home staffing levels and standards to regulatory deficiencies. J Gerontol. (2009) 64B:269–78. 10.1093/geronb/gbn019 19181692 PMC2655170

[32] Zhang NJ Wan TTH. Effects of institutional mechanisms on nursing home quality. J Health Hum Serv Adm. (2007) 29:380–408. 17571466

[33] Pittman T. Care deficiencies and super-organization of American nursing homes in hospital referral region: an ecological cohort study, 08 June 2020, PREPRINT (Version 1) available at Research Square. 10.21203/rs.3.rs-30219/v1 PMC7854530 33553087

