# Corrigendum: Care deficiencies and super-organization of American nursing homes in hospital referral region

**DOI:** 10.3389/fpubh.2024.1428751

**Published:** 2024-07-29

**Authors:** Tyler Pittman

**Affiliations:** Biostatistics Department, Princess Margaret Cancer Centre, Toronto, ON, Canada

**Keywords:** social network analysis, degree-based centrality, ownership group, total weighted health survey score, registered organization

In the published article, there was an error. Effect medication should be displayed as effect modification.

A correction has been made to **Methods**, *Facility and Resident Characteristic Measures in Nursing Homes*, Paragraph 1. This sentence previously stated:

“Effect medication between ownership type and affiliation class of nursing home was also explored.”

The corrected sentence appears below:

“Effect modification between ownership type and affiliation class of nursing home was also explored.”

In the published article, there was an error. AHH should be displayed as AHHI.

A correction has been made to **Methods**, *Measures Denoting Super-Organization in*

*Focal Areas*, Paragraph 4. This sentence previously stated:

“The delta Herfindahl-Hirschman Index (HHI) is the difference between the AHH and the HHI (4).”

The corrected sentence appears below:

“The delta Herfindahl-Hirschman Index (HHI) is the difference between the AHHI and the HHI (4).”

In the published article, there was an error. Effect medication should be displayed as effect modification.

A correction has been made to **Discussion**, Paragraph 3. This sentence previously stated:

“Effect medication between ownership type and super-organization of nursing home was shown in the association to care deficiencies.”

The corrected sentence appears below:

“Effect modification between ownership type and super-organization of nursing home was shown in the association to care deficiencies.”

The authors apologize for these errors and state that this does not change the scientific conclusions of the article in any way. The original article has been updated.

